# GDS score as screening tool to assess the risk of impact of chronic conditions and depression on quality of life in hospitalized elderly patients in internal medicine wards

**DOI:** 10.1097/MD.0000000000026346

**Published:** 2021-07-02

**Authors:** Christiano Argano, Nicola Catalano, Giuseppe Natoli, Marika Lo Monaco, Salvatore Corrao

**Affiliations:** a Department of Internal Medicine, National Relevance and High Specialization Hospital Trust ARNAS Civico, Di Cristina, Benfratelli; b Dipartimento di Promozione della Salute, Materno Infantile, Medicina Interna e Specialistica di Eccellenza “G. D’Alessandro”, PROMISE, University of Palermo, Palermo, Italy.

**Keywords:** depression, frail elders, multiple chronic conditions, quality of life

## Abstract

Aging of population is characterized by multiple chronic conditions in the same individual. Health-related quality of life (HR-QOL) reflects the multidimensional impact of chronic disease on population and it is increasingly analysed as outcomes.

The aim of this study was the evaluation of the predictors of quality of life among elderly patients hospitalized in internal medicine ward, investigating the effect of comorbidities on health-related quality of life.

Data collected in this cross-sectional study were analysed. Socio-demographic, clinical characteristics, disease distribution and quality of life by the 12-Item Short Form Health Survey (SF-12) were evaluated.

Of 240 inpatients, subjects with Barthel Index (BI)≤40 were 23.7%, 55% had a Geriatric Depression Scale (GDS)≥2. After categorizing mental component score (MCS) and physical component score (PCS) in five classes, we found that diabetics and patients with cancer were more frequent in the first class of MCS while patients with NYHA III-IV are significantly more frequent in the first class of PCS. When we classified patients according to GDS≥2 or < 2, subjects with GDS≥2 had BI and MCS significantly lower. In the multivariate analysis GDS score ≥2 was independently associated with first MCS class [16.32 (3.77–70.68)] while NYHA III-IV class and claudicatio intermittents were strong predictors of the worst PCS class [9.54 (1.97–47.40), 2.53 (1.16–5.49), respectively]. Liver disease was independently associated with GDS≥2 [5.26 (1.13–24.39)].

Our study highlighted the impact of chronic diseases on health-related quality of life in elderly subjects hospitalized in an internal medicine ward pointing out the importance of taking into account patient's needs and perception and the setting up of a personalised health-care. Patients with diabetes and liver disease along with persons affected by cancer need psychological support to improve their quality of life. A GDS score ≥ 2 is a strong predictor of poor quality of life and should trigger an in-depth assessment of mental health in this kind of patients.

## 1 Introduction

Advances in public health and medical practice, have determined the worldwide increasing of life expectancy. According to the 2018 report by the Academy of Medical Sciences, globally, by 2050, people aged 60 years and older will reach 2.1 billion.^[1]^ In Europe by 2060, people aged 65 and older will rise from 18% to 28% of the whole population and the share of people aged 80 and older will rise from 5% to 12% just like young person.^[2]^ The same trend is expected in Italy, where by 2050, people aged 65 and older it is estimated to be 24% of the population and 15% of them is projected to be older than 80 years.^[3]^ As a result of this demographic change, the impact of chronic diseases was most likely to occur^[4]^ and many people are living with more than one conditions, and are more complex patient. In clinical practice patient complexity is defined by the interaction among medical issues, psychological and social characteristics and healthcare factors.^[5]^ In view of this, a comprehensive assessment is necessary. Health related quality of life (HRQOL) is an instrument useful to characterize the impact of multiple chronic conditions on population by exploring patient's physical, emotional, and social functioning.^[6]^ According to World Health Organization quality of life is defined as “an individual's perception of their position in life in the context of the culture and value systems in which they live and in relation to their goals, expectations, standards and concerns”.^[7]^ The association between chronic diseases and HRQOL has also been well studied^[8]^ in particular the effect of multiple chronic conditions on HRQOL.^[9,10]^ Although this piece of evidence, few data are available about the association between chronic conditions and HRQOL in patients admitted to internal medicine wards.^[11]^ Given this background the aim of this investigation was to evaluate the predictors of quality of life in a cross-sectional study about elderly patients hospitalized in internal medicine ward focusing on the effect of comorbidities on health-related quality of life.

## 2 Patients and methods

Two hundred and forty elderly inpatients were consecutively enrolled. The patients were admitted to the Department of Internal Medicine, National Relevance and High Specialization Hospital Trust ARNAS Civico, Di Cristina, Benfratelli of Palermo (Italy). Each patient gave informed consent after receiving a detailed description of the study procedures. This cross-sectional study was approved by the Ethics Committee of our institution. Socio-demographic variables were considered. The following clinical characteristics were evaluated: disease distribution at hospital admission (classification was based on the International Classification of Diseases-Ninth Revision), performance in basic activities of daily living (measured by means of the Barthel Index), comorbidity and severity indexes (according to the Cumulative Illness rating Scale CIRS-c and CIRS-s), cognitive status according to the Short Blessed Test, SBT, and mood disorders using the Geriatric Depression Scale, GDS score.^[12]^ Moreover, we subdivided subjects according to low risk and high risk of depression.

### 2.1 Quality of life (QOL)

Quality of life was measured by the 12-Item Short Form Health Survey (SF-12), developed for the Medical Outcomes Study (MOS), a multi-year study of patients with chronic conditions.^[13,14]^ The SF-12 consists of 12 items from the larger SF-36 and it is used to measure health status in general population. The SF-12 measures physical functioning, role limitations due to physical health problems, bodily pain, general health, vitality (energy/fatigue), social functioning, role limitations due to emotional problems, and mental health (psychological distress and psychological well-being). Two composite scores— the physical component score (PCS) and the mental component score (MCS) are computed from all 12 items using a standard scoring algorithm.^[15]^ The PCS score primarily focuses on physical functioning, role-physical, bodily pain, general health and vitality scales. The MCS focuses on vitality, social functioning, role-emotional, and emotional well-being scales. Both PCS and MCS scores range from 0 to 100, higher score indicates better health status.^[16]^ All patients were categorized according to PCS and MCS. To simplify the analysis, we subdivided PCS and MCS score in five classes comparing the worst class to the other ones.

### 2.2 Statistical analysis

Data were reported as percentages for categorical variables and as means (95% confidence intervals) for quantitative variables. A Barthel index (BI) score of ≤ 40 was used to recognize patients with significant disability according to our population characteristics and it is the best cut-off value in prediction of mortality.^[17]^ The comparison between groups was made using the exact Fisher test for contingency tables and the z test for comparison of proportions. The non-parametric Mann–Whitney *U* test was used for comparison of quantitative variables. Multivariate logistic analysis was used to explore the relationship between variables and outcomes. Odds ratios (ORs) and 95% confidence intervals (95%CIs) were computed. The choice of variables was performed according to the Hosmer–Lemeshow methodology:^[18]^ after univariate analysis, only variables with a *P* < .20 were included in the final model; then, through a backward process, variables were excluded until a significance level of *P* < .05 was reached for each variable. A two-tailed *P* < .05 was considered statistically significant. Stata (StataCorp. 2016. Stata Statistical Software: Release 14.1. College Station, TX: StataCorp LP) was used for database management and analysis.

## 3 Results

**Figure 1:**
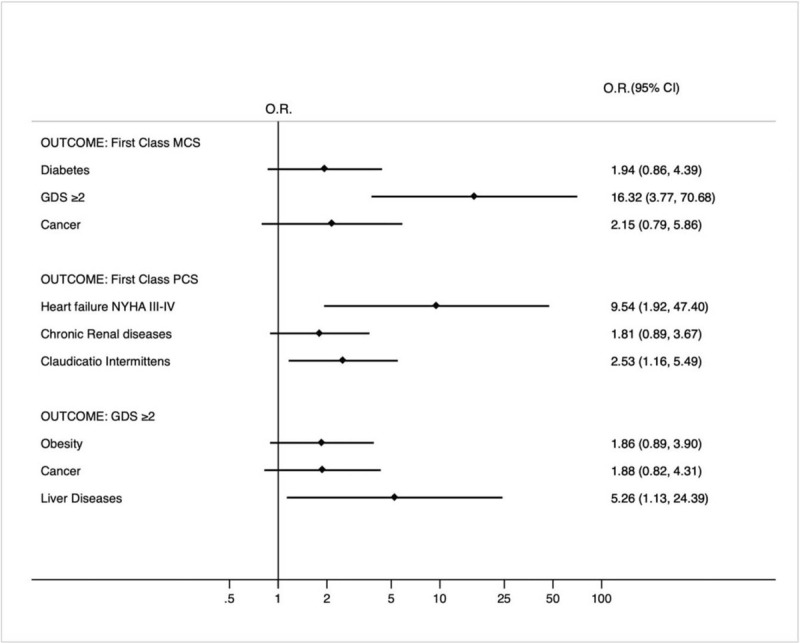
Multivariate analysis according to first class MCS, first class PCS and GDS ≥ 2 (high risk of depression) (OR = odds ratio; 95% CI = 95% confidence interval). The variables reported in the three models were selected by the Hosmer–Lemeshow methodology; only variables with a *P* < .20 are shown in the graph. ∗GDS = Geriatric depression score, MCS =Mental component score, PCS = Physical component score.

Table 1 shows baseline characteristics of hospitalized inpatients. Among patients 52.1% were male, the mean age was 76.4 (75.3–77.4) years old and the mean BMI was 27.32 (26.63–28.02). Current smokers and ex-smokers were 43.3%, 50.4% of patients were diabetics. In-patients with clinically significant disability (Barthel index ≤ 40) were 23.7% and 12.1% had a Barthel index < 20. More than half of subjects had a probable depression (GDS score ≥ 2), 43.7% had a Short-Blessed Test > 10. Mean severity and co-morbidity index were 2.8 and 2.9, respectively. Mean MCS and PCS were 42.8 and 34.9 respectively. Overall, disease distribution showed that diabetes, arterial hypertension, acute infections, chronic renal failure, obesity, COPD and acute anaemia were more frequent (Fig. 1). After dividing the MCS and PCS score in five classes, we analyse the difference. A significantly higher proportion of diabetics was present in the first class of MCS (Table 2) in comparison with the other classes (*P* = .05) as well as patients with cancer (*P* = .03). Patients in the first class MCS had a Barthel Index score (*P* = .0138) and a physical component score (*P* = .0244) significantly lower, a GDS significantly higher (*P* < .0001) along with a higher rate of people with GDS ≥ 2. Taking into account the PCS (Table 3), the proportion of inpatients with NYHA III-IV class is significantly higher in the first class of PCS in comparison with the other ones (*P* = .0026). People in the first-class PCS were older (78.2 vs 75.2 years, *P* = .0110), 41.5% were male. They had a Barthel index score significantly lower (*P* < .0001) with a share of subjects with a Barthel Index Score < 40 and < 20 significantly higher in comparison with the other classes (*P* = .0060 and *P* = .0027 respectively). A Geriatric Depression Scale significantly higher (*P* = .0004) along with a percentage of patients with a GDS ≥ 2 (*P* = .0098) was present in the first class MCS. In view of the evidence that 55% of subjects had a GDS score ≥ 2 we divided population according to low risk of depression and high risk of depression (Table 4). Patients with a high risk for depression had a Barthel index and MCS significantly lower (*P* < .0001 for both comparisons) along with a PCS (*P* = .0347). In-patients with a GDS score ≥ 2 had a Short-Blessed Test > 10 significantly higher (.0012). A significantly higher proportion of patients with liver diseases was present in subjects with high risk of depression (*P* = .0004 and *P* = .0171), respectively. Regarding drug therapy, pump inhibitors, heparins, diuretics, antibiotics, antiaggregating agents and insulins were the most drugs taken in subjects with high risk of depression (89.8%, 55%, 50.7, 50.7%, 42.5%, 42% respectively). In patients with low risk of depression pump inhibitors, diuretics, antibiotics, heparins, beta blockers and antiaggregating agents (74.2%, 43.5%, 43.5%, 41.6%, 34.6%, 31.7% respectively) were given more frequent. When we assessed independent predictors of lower MCS class by a multivariate analysis (Fig. 1), GDS score ≥ 2 was independently associated with first MCS class I. Regarding first PCS class, heart failure NYHA III-IV class and claudicatio intermittents were strongly independently associated with the worst PCS class. Liver disease was the only predictor of probable depression.

**Table 1:** Patient socio-demographic, laboratory and clinical baseline characteristics of hospitalized inpatients.

N	240
Age (yrs)^∗^	76.4 (75.3–77.4)
Male (%)	52.1
Body Mass Index (BMI)^∗^	27.32 (26.63–28.02)
Current smokers (%)	9.6
Ex smokers (%)	33.7
Diabetes mellitus (%)	50.4
Type 1 diabetes (%)	1.7
Type 2 diabetes (%)	48.3
Systolic pressure (mmHg)^∗^	129.7 (127.3–132.1)
Diastolic pressure (mmHg)^∗^	73.8 (72.6–75.1)
Framingham risk > 10% (%)	57.9
ABI (right)^∗^	1.00 (0.97–1.02)
ABI (left)^∗^	1.02 (1.00–1.04)
Barthel index^∗^	65.6 (61.7–69.6)
Barthel index < 20 (%)	12.1
Barthel index < 40 (%)	23.7
Geriatric Depression Scale (GDS)^∗^	1.8 (1.6–2.0)
GDS ≥2 (%)	55.0
Short Blessed Test (SBT)^∗^	9.8 (8.8–10.7)
SBT > 10 (%)	43.7
Severity index (CIRS)^∗^	2.8 (2.7–2.9)
Comorbidity index (CIRS)^∗^	2.9 (2.6–3.2)
Mental Component Score (MCS)^∗^	42.8 (41.4–44.3)
Physical Component Score (PCS)^∗^	34.9 (33.8–36.0)
Glycemia (mg/dl)^∗^	119.12 (109.53–128.71)
Blood urea nitrogen (mg/dL)^∗^	27.10 (24.91–29.29)
Creatinine (mg/dL)^∗^	2.52 (0.84–4.20)
Total Cholesterol (mg/dL)^∗^	155.42 (149.22–161.63)
HDL (mg/dL)^∗^	38.31 (36.45 – 40.18)
Triglycerides (mg/dL)^∗^	127.02 (114.90–139.14)
Hemoglobin (g/dL)^∗^	11.3 (11.1–11.6)
Diabetes (%)	50.4
Hypertension (%)	41.7
Acute infections (%)	26.7
Chronic renal disease (%)	19.2
Obesity (%)	17.1
COPD (%)	15.8
Acute anemia (%)	15.0
Cancer (%)	12.5
Chronic anemia (%)	11.2
Atrial fibrillation (%)	8.7
Dilated cardiomyopathy (%)	7.5
Liver diseases (%)	7.1
Osteoporosis (%)	6.2
Heart failure NYHA I-II (%)	5.4
Heart failure NYHA III-IV (%)	4.6
Peripheral artery disease (%)	4.2
Ischemic stroke (%)	1.2
Myocardial infarction (%)	0.4

∗ Data are reported as mean (95% Confidence Interval). ABI = Ankle-Brachial Index, BMI = Body Mass Index, CIRS = Cumulative Illness Rating Scale, COPD = Chronic obstructive pulmonary disease, GDS = Geriatric Depression Scale, HDL = High Density Lipoproteins, NYHA = New York Heart Association, SBT = Short Blessed Test.

**Table 2:** Patient socio-demographic, laboratory and clinical baseline characteristics of hospitalized inpatients according to first class in comparison to other classes of Mental Component Score (MCS).

Variables	I Class N = 34	II-V Classes N = 197	*P* =
Age (yrs)^∗^	76.1 (73.8–78.4)	76.3 (75.1–77.5)	.9235
Male (%)	52.9	50.8	.8144
Body Mass Index (BMI)^∗^	27.97 (26.30–29.63)	27.17 (26.40–27.94)	.2477
Current smokers (%)	8.8	8.6	.9704
Ex smokers (%)	38.2	33.5	.5911
Type 1 diabetes (%)	2.9	1.5	.5582
Type 2 diabetes (%)	61.8	44.7	.0652
Systolic pressure (mmHg)^∗^	134.2 (127.4–141.1)	128.5 (125.9–131.2)	.1638
Diastolic pressure (mmHg)^∗^	77.5 (73.7–81.2)	72.9 (71.6–74.3)	**.0251**
Framingham risk > 10% (%)	55.9	58.9	.7430
ABI (right)^∗^	0.99 (0.94–1.05)	1.00 (0.97–1.03)	.8070
ABI (left)^∗^	0.99 (0.95–1.03)	1.02 (1.00–1.05)	.3644
Barthel index^∗^	55.7 (45.4–65.9)	68.2 (63.9–72.4)	**.0138**
Barthel index < 20 (%)	20.6	10.1	.0803
Barthel index < 40 (%)	29.4	21.8	.3314
Geriatric Depression Scale (GDS)^∗^	3.03 (2.72–3.33)	1.59 (1.40–1.78)	**<.0001**
GDS ≥2 (%)	93.9	48.2	**<.0001**
Short Blessed Test (SBT)^∗^	11.7 (9.0–14.3)	9.4 (8.3–10.5)	.1049
SBT > 10 (%)	41.2	59.2	**.0513**
Severity index (CIRS)^∗^	2.59 (2.20–2.99)	2.82 (2.67–2.97)	.1223
Comorbidity index (CIRS)^∗^	2.58 (1.81–3.36)	2.95 (2.64–3.25)	.3510
Physical Component Score (PCS)^∗^	32.13 (29.34–34.92)	35.34 (34.16–36.52)	**.0244**
Glycemia (mg/dl)^∗^	126.2 (103.8–148.5)	118.0 (107.5–128.5)	.3057
Blood urea nitrogen (mg/dL)^∗^	25.1 (19.3–31.0)	27.8 (25.4–30.3)	.2027
Creatinine (mg/dL)^∗^	1.23 (0.85–1.62)	2.82 (0.77–4.86)	.4721
Hemoglobin (g/dL)^∗^	10.8 (10.2–11.4)	11.3 (11.0–11.7)	.2771
Diabetes (%)	64.7	46.7	**.05**
Hypertension (%)	32.3	42.6	.2603
COPD (%)	26.5	14.7	.0879
Cancer (%)	23.5	10.7	**.0365**
Obesity (%)	20.6	15.2	.4313
Atrial fibrillation (%)	20.6	16.7	.585
Chronic renal disease (%)	11.8	20.8	.2187
Chronic anemia (%)	8.8	10.6	.7459
Acute anemia (%)	8.8	16.2	.2651
Liver diseases (%)	8.8	7.1	.7233
Acute infections (%)	2.9	11.7	.1232
Heart failure NYHA III-IV (%)	2.9	4.6	.6668
Ischemic stroke (%)	2.9	1.0	.3597
Myocardial infarction (%)	0.0	0.5	.6772
Dilated cardiomyopathy (%)	0.0	8.1	.085
Osteoporosis (%)	0.0	7.6	.0961
Heart failure NYHA I-II (%)	0.0	6.6	.1231
Peripheral artery disease (%)	0.0	4.6	.2036

∗ Data are reported as mean (95% Confidence Interval). ABI = Ankle-Brachial Index, BMI = Body Mass Index, CIRS = Cumulative Illness Rating Scale, COPD = Chronic obstructive pulmonary disease, GDS = Geriatric Depression Scale, NYHA = New York Heart Association, SBT = Short Blessed Test.

**Table 3:** Patient socio-demographic, laboratory and clinical baseline characteristics of hospitalized inpatients according to first class in comparison to other classes of Physical Component Score (PCS).

Variables	I Class N = 82	II-V Classes N = 149	*P* =
Age (yrs)^∗^	78.2 (76.5–80.0)	75.2 (73.9–76.5)	**.0110**
Male (%)	41.5	56.4	**.0300**
Body Mass Index (BMI)^∗^	27.20 (26.11–28.29)	27.33 (26.42–28.25)	.7428
Current smokers (%)	6.1	10.1	.3046
Ex smokers (%)	23.2	40.3	**.0088**
Type 1 diabetes (%)	1.2	2.0	.6580
Type 2 diabetes (%)	48.8	46.3	.7188
Systolic pressure (mmHg)^∗^	130.7 (126.8–134.6)	128.6 (125.4–131.8)	.5928
Diastolic pressure (mmHg)^∗^	74.2 (72.6–75.8)	72.5 (70.2–74.7)	.1727
Framingham risk > 10% (%)	52.4	61.7	.1697
ABI (right)^∗^	1.01 (0.97–1.04)	0.99 (0.96–1.03)	.8234
ABI (left)^∗^	1.00 (0.97–1.03)	1.03 (1.00–1.06)	.2853
Barthel index^∗^	54.15 (47.38–60.92)	73.09 (68.51–77.66)	**<.0001**
Barthel index < 20 (%)	19.5	7.4	**.0060**
Barthel index < 40 (%)	34.1	16.8	**.0027**
Geriatric Depression Scale (GDS)^∗^	2.25 (1.94–2.56)	1.56 (1.35–1.78)	**.0004**
GDS ≥2 (%)	66.7	48.6	**.0098**
Short Blessed Test (SBT)^∗^	10.46 (8.91–12.00)	9.44 (8.16–10.71)	.1019
SBT > 10 (%)	51.3	59.2	.2553
Severity index (CIRS)^∗^	2.66 (2.44–2.88)	2.84 (2.67–3.02)	.3704
Comorbidity index (CIRS)^∗^	2.86 (2.29–3.43)	2.92 (2.59–3.25)	.9057
Mental Component Score (MCS)^∗^	41.19 (38.30–44.08)	43.74 (42.19–45.29)	.0858
Glycemia (mg/dl)^∗^	108.4 (97.3–119.5)	123.0 (110.6–135.5)	.4792
Blood urea nitrogen (mg/dL)^∗^	27.9 (24.3–31.4)	27.2 (24.3–30.1)	.4449
Creatinine (mg/dL)^∗^	1.21 (1.01–1.41)	3.33 (0.65–6.01)	.9405
Hemoglobin (g/dL)^∗^	11.2 (10.8–11.7)	11.3 (10.9–11.7)	.9287
Diabetes (%)	51.2	48.3	.6734
Hypertension (%)	40.2	41.6	.8399
Chronic renal disease (%)	24.4	16.8	.1622
Atrial fibrillation (%)	20.7	15.4	.3088
Obesity (%)	20.6	15.2	.4313
COPD (%)	17.1	16.1	.8497
Acute anemia (%)	15.8	14.8	.8252
Cancer (%)	14.6	11.4	.4791
Chronic anemia (%)	11.0	10.1	.8286
Heart failure NYHA III-IV (%)	9.8	1.3	**.0026**
Acute infections (%)	8.5	11.4	.4935
Liver diseases (%)	8.5	6.7	.6112
Dilated cardiomyopathy (%)	7.3	6.7	.8623
Osteoporosis (%)	4.9	7.4	.4598
Heart failure NYHA I-II (%)	2.4	7.4	.1187
Peripheral artery disease (%)	1.2	5.4	.1189
Myocardial infarction (%)	0.0	0.7	.4572
Ischemic stroke (%)	0.0	2.0	.1959

∗ Data are reported as mean (95% Confidence Interval). ABI = Ankle-Brachial Index, BMI = Body Mass Index, CIRS = Cumulative Illness Rating Scale, COPD = Chronic obstructive pulmonary disease, GDS = Geriatric Depression Scale, NYHA = New York Heart Association, SBT = Short Blessed Test.

**Table 4:** Patient socio-demographic, laboratory and clinical baseline characteristics of hospitalized inpatients according to the risk of depression.

Variables	High risk for depression (GDS ≥ 2) N = 127	Low risk for depression (GDS <2) N = 104	*P*
Age (yrs)^∗^	76.3 (74.9–77.7)	76.4 (74.7–78.1)	.9502
Male (%)	50.4	51.9	.8170
Body Mass Index (BMI)^∗^	27.37 (26.51–28.24)	27.52 (26.37–28.67)	.4910
Current smoking (%)	11.8	7.7	.2983
Ex smokers (%)	36.2	33.6	.6842
Diabetes mellitus (%)	52.0	48.1	.5562
Type 1 diabetes (%)	3.1	0.0	.0679
Type 2 diabetes (%)	48.0	48.1	.9945
Systolic pressure (mmHg)^∗^	130.9 (127.8–133.9)	127.9 (123.9–131.9)	.6011
Diastolic pressure (mmHg)^∗^	74.8 (73.2–76.5)	72.5 (70.5–74.5)	.0566
Framingham risk > 10% (%)	55.9	63.5	.2448
ABI (right)^∗^	1.00 (0.96–1.04)	1.01 (0.98–1.04)	.5231
ABI (left)^∗^	1.01 (0.98–1.05)	1.04 (1.01–1.07)	.1424
Barthel index^∗^	60.3 (55.3–65.4)	74.9 (69.4–80.3)	**<.0001**
Barthel index < 20 (%)	11.8	7.7	.2983
Barthel index < 40 (%)	26.0	17.3	.1137
Short Blessed Test (SBT)^∗^	10.8 (9.5–12.1)	8.1 (6.8–9.5)	**.0031**
SBT > 10 (%)	52.8	31.4	**.0012**
Severity index (CIRS)^∗^	2.9 (2.7–3.1)	2.7 (2.5–2.8)	.1128
Comorbidity index (CIRS)^∗^	3.2 (2.7–3.6)	2.6 (2.2–3.0)	.0690
Mental Component Score (MCS)^∗^	39.6 (37.6–41.5)	46.8 (44.8–48.7)	**<.0001**
First Class MCS (%)	25.2	2.0	**<.0001**
Physical Component Score (PCS)^∗^	33.8 (32.4–35.3)	36.3 (34.6–37.9)	**.0347**
First Class PCS (%)	42.3	25.7	**.0098**
Glycemia (mg/dl)^∗^	119.7 (104.1–135.3)	118.4 (107.3–129.5)	.7044
Blood urea nitrogen (mg/dL)^∗^	26.9 (24.0–29.9)	27.7 (24.2–31.1)	.5739
Creatinine (mg/dL)^∗^	1.22 (1.06–1.39)	4.27 (0.40–8.14)	.7220
Hemoglobin (g/dL)^∗^	11.1 (10.8–11.4)	11.3 (10.9–11.8)	.6361
Diabetes (%)	52.0	48.1	.5562
Hypertension (%)	46.5	37.5	.1706
Acute infections (%)	27.6	27.9	.9561
Chronic renal disease (%)	22.0	13.5	.0923
Obesity (%)	20.5	12.5	.1076
Acute anemia (%)	16.5	12.5	.3891
COPD (%)	15.7	17.3	.7504
Cancer (%)	15.7	9.6	.1677
Atrial fibrillation (%)	10.2	7.7	.5034
Liver diseases (%)	9.4	1.9	**.0171**
Chronic anemia (%)	8.7	11.5	.4675
Dilated cardiomyopathy (%)	8.7	6.7	.5860
Osteoporosis (%)	6.3	6.7	.8946
Heart failure NYHA III-IV (%)	6.3	2.9	.2253
Heart failure NYHA I-II (%)	5.5	5.8	.9327
Peripheral artery disease (%)	3.1	5.8	.3304
Ischemic stroke (%)	0.8	1.9	.4482
Myocardial infarction (%)	0.8	0.0	.3645

∗ Data are reported as mean (95% Confidence Interval). ABI = Ankle-Brachial Index, BMI = Body Mass Index, CIRS = Cumulative Illness Rating Scale, COPD = Chronic obstructive pulmonary disease, GDS = Geriatric Depression Scale, MCS = Mental Component Score, NYHA = New York Heart Association, PCS = Physical Component Score, SBT = Short Blessed Test.

### 3.1 Discussion

The present study is one of very few performed in internal medicine ward. Because of the demographic change due to the aging of the population, the management of multiple chronic conditions are becoming the main challenge in clinical practice. Along with this issue, in subjects admitted to internal medicine wards the assessment of the impact of chronic diseases on health-related quality of life is important in order to identify subjects at higher risk of poor health and quality of life and to improve the quality of health-care. To date few data are available about the perception of health from in-patients admitted to internal medicine wards.^[11]^ Different studies showed that chronic medical conditions affect physical functioning and reduced quality of life.^[19,20]^ Particularly, gastrointestinal diseases, cerebrovascular conditions, musculoskeletal and renal diseases determined poorer quality of life.^[20]^

One of the main finding of our analysis concerned the impact of heart failure and intermittent claudication on physical component of HRQOL. This is likely to be closed linked to the functional limitation with a progressive reduction of daily activities.^[21]^ In fact, heart failure had a negative impact on quality of life with increasing functional class, by a significant association with physical functioning and psychosocial function. Our results are consistent with previous studies that showed that cardiovascular diseases had a significant impact on physical component in elderly patients.^[22]^ In addition, intermittent claudication has a significant reduction in health-related quality of life (HRQoL) caused by impaired mobility and by cardiovascular morbidity. Patients with peripheral artery disease had a significant impairment of physical functional score in comparison with controls^[23,24]^ and even worse scores than individuals with coronary artery disease and congestive heart failure.^[25]^

Our findings indicate that GDS ≥ 2 was an independent strong predictor of first class MCS. It is well known that depression is an important determinant of quality of life. Mood and anxiety disorders are the most prevalent among elderly patients.^[26]^ As shown by Beladon et al^[22]^ a strong relationship between depression symptoms and mental quality of life is observed in older primary care patients both on men and women. Mood disorders had the greatest impact on HRQoL among the elderly.^[22,27,28]^ Mainly, depression affects emotion, motivation, and cognition that are closely related to vitality, role function, or social functioning, some of the major components of HRQoL measures.^[29]^ Data of community-dwelling elderly subjects, showed that GDS scores above 9/30 or 5/15 were able to predict subjects in whom treatment could likely improve quality of life.^[30]^

A recent analysis of the DIAREG registry, a nationwide German general medicine practice database showed that patients with <2 years diabetes duration had a significantly decreased of mental component score.^[31]^ Moreover, data from the longitudinal Living with Diabetes Study showed that depression and anxiety were associated with poorer diabetes-specific quality of life.^[32]^ Wong et al showed that insulin treatment was associated with lower MCS-12.^[33]^ In the present study diabetes did not enter the multivariate analysis as independent predictor of MCS, although diabetes was suggestive of worst mental component score. A possible explanation for the apparent discrepancy lies in the lack of information about the diabetic disease duration, the limited sample size and type of drugs taken.

Cancer diagnosis often results in negative impacts on health-related quality of life^[34]^ because of alteration of mental health, anxiety, fear of cancer recurrence,^[35]^ therefore the risk of depression is more likely among subjects with cancer.^[36]^ Depression in people affected by cancer is negatively associated with health-related quality of life.^[37,38]^ Ehus et al showed that the diagnosis of small breast cancer affects health related quality of life including both physical and mental component score.^[39]^ Our data are in accordance with this piece of evidence even if a cancer did not represent a strong predictor of lower mental component score and GDS ≥ 2 probably due to the limited sample size.

It is well known that depression and cognitive impairment lead to progressive disability,^[40,41]^ especially in oldest-old subjects^[42]^ with implications on short and long-term outcomes.

Globally, depression represents the leading cause of disability.^[43]^ Depression combined with other chronic diseases result in greater reduction of health-related quality of life (HRQoL).^[44]^ The present analysis highlighted the role of liver diseases that significantly increased the risk of depression. According to the Global Burden of Diseases, Injuries, and Risk Factors Study, the combination of depression and alcohol abuse lead to lifespan disability and premature mortality rates.^[45,46]^ Subjects with NAFLD and HCV have a higher prevalence of depression in comparison with HBV patients and the general population.^[47]^ Recent data showed that in subjects with major depressive syndrome a significantly higher prevalence and incidence of chronic liver disease than the general population is detectable.^[48]^ In this population-based database, an older age, the male sex, diabetes, hyperlipidemia and first-generation antipsychotic use were associated with chronic liver disease.

In this study some limitations have to be outlined. First of all, this is a single centre analysis. No history data were available about timing of comorbidities to assess possible relationship with both quality of life measures and depression. No other specific instrument for assessing symptoms of depression except for geriatric depression scale was used. No investigation about any correspondence between different type of diseases and outcomes by specific treatment was made. The population study consists only of oldest old patients and the sample size is limited. On the contrary, the major strength of our study lies in the rigorous collection of data in a standardized fashion. Although the sample size is limited the results are quite robust to support further research. However, our data may not be generalizable. Our observations need to be verified by later studies to avoid population bias due to the single centre study. Finally, our analysis revealed the importance of GDS that is a quick and simple tool that might be utilized in every day clinical practice of internal medicine wards.

## 4 Conclusion

This analysis showed the impact of chronic diseases on health-related quality of life in the real-world scenario of an internal medicine ward. This is the first study that pointed out the association between GDS score and chronic conditions in elderly inpatients. Thus, GDS score might be used as a screening tool to select elderly patients that must be assessed for psychological and therapeutic support in internal medicine ward. Our study strongly supports a different approach to subjects admitted to internal medicine ward that take into account of patient's needs and perception. This could be the first step in order to reduce health care costs, the burden of hospitalization and to establish a personalized care. Further studies are necessary to confirm our data in multicentre reliability and a bigger sample size.

## Author contributions

**Conceptualization:** Salvatore Corrao, Christiano Argano, Nicola Catalano.

**Data curation:** Giuseppe Natoli.

**Formal analysis:** Salvatore Corrao, Christiano Argano, Nicola Catalano.

**Investigation:** Salvatore Corrao, Nicola Catalano, Giuseppe Natoli, Marika Lo Monaco.

**Methodology:** Salvatore Corrao, Christiano Argano, Giuseppe Natoli.

**Project administration:** Salvatore Corrao, Christiano Argano, Giuseppe Natoli.

**Resources:** Salvatore Corrao.

**Software:** Giuseppe Natoli.

**Supervision:** Salvatore Corrao, Christiano Argano.

**Validation:** Salvatore Corrao, Christiano Argano, Nicola Catalano, Giuseppe Natoli, Marika Lo Monaco.

**Visualization:** Salvatore Corrao, Christiano Argano, Nicola Catalano, Giuseppe Natoli, Marika Lo Monaco.

**Writing – original draft:** Christiano Argano.

**Writing – review & editing:** Salvatore Corrao, Giuseppe Natoli.
